# When Less Is More: Evolutionary Origins of the Affect Heuristic

**DOI:** 10.1371/journal.pone.0046240

**Published:** 2012-10-03

**Authors:** Jerald D. Kralik, Eric R. Xu, Emily J. Knight, Sara A. Khan, William J. Levine

**Affiliations:** Department of Psychological and Brain Sciences, Dartmouth College, Hanover, New Hampshire; CNR, Italy

## Abstract

The human mind is built for approximations. When considering the value of a large aggregate of different items, for example, we typically do not summate the many individual values. Instead, we appear to form an immediate impression of the likeability of the option based on the average quality of the full collection, which is easier to evaluate and remember. While useful in many situations, this affect heuristic can lead to apparently irrational decision-making. For example, studies have shown that people are willing to pay more for a small set of high-quality goods than for the same set of high-quality goods with lower-quality items added [e.g. 1]. We explored whether this kind of choice behavior could be seen in other primates. In two experiments, one in the laboratory and one in the field, using two different sets of food items, we found that rhesus monkeys preferred a highly-valued food item alone to the identical item paired with a food of positive but lower value. This finding provides experimental evidence that, under certain conditions, macaque monkeys follow an affect heuristic that can cause them to prefer less food. Conservation of this affect heuristic could account for similar ‘irrational’ biases in humans, and may reflect a more general complexity reduction strategy in which averages, prototypes, or stereotypes represent a set or group.

## Introduction

Many real-world problems that humans solve effortlessly appear intractable when analyzed computationally [2], [3], [4]. We solve these ‘intractable’ problems through the use of heuristics. Our mind replaces complicated problems with simpler ones that engender easy and reasonable approximations [4], [5], [6], [7], [8], [9], [10]. For example, the *affect heuristic* provides an immediate evaluation of stimuli based on positive or negative valence [11]. Evidence has amassed for the significance of affect in judgment and decision-making [4], [5], [11], [12], [13], [14], [15], [16], [17], [18], [19], [20], [21], [22], [23], leading Kahneman [5] to state that, “The idea of an affect heuristic…is probably the most important development in the study of…heuristics in the past few decades. There is compelling evidence for the proposition that every stimulus evokes an affective evaluation, which is not always conscious…(pp. 710).”

An affective evaluation process would be considered a heuristic if there are cases in which only some stimulus attributes are evaluated, while others are neglected. A phenomenon that demonstrates this is the *less-is-more effect* [11]. For example, when considering the overall value of a large number of different items, we normally do not painstakingly summate the individual values. Instead, we appear to form an immediate impression of the attractiveness of the option based on the average quality, which is easier to evaluate and remember [1], [4], [5], [11], [17], [24], [25], [26], [27]. Thus, in one study, participants rated a 24-piece dinnerware set more highly than one with the same twenty-four pieces, plus sixteen more pieces that included nine broken ones (Hsee, 1998). Although the latter option offered the same twenty-four pieces (with an additional seven intact), its attractiveness was reduced by the additional broken items. A similar less-is-more effect has been found in many fields of study, under different experimental conditions, and with various items: e.g., from questions involving saving lives and earning money, to ratings of clothing, ice cream, and baseball cards [1], [4], [5], [11], [24], [26], [27], [28].

The affect heuristic is limited by how readily stimulus attributes can be mapped to a point on a one-dimensional valence scale. That is, the heuristic will normally be applied to attributes that are readily evaluable as good or bad, such as item quality (are broken dishes bad?), and will not be applied to attributes that are difficult to evaluate, such as absolute quantity (exactly how good or bad is 24 of something?) [4], [6], [7], [11], [25], [26], [27], [28]. Thus, when evaluating the dinnerware, quantity is neglected, and the lower quality items reduce the appeal of the entire set.

There is also evidence that the affect heuristic can be overridden when the logical choice can be seen clearly, as with direct comparisons. Thus, people tend to use the heuristic when rating experiences or options *separately* but not when they are allowed to compare them *directly*. In the latter case, people can see that the preferred items are contained in the option with the larger number of items and tend to make the logical choice [26], [27], [28].

Because many judgment and decision-making heuristics and biases appear universal and difficult to overcome, they may have an innate component that is shared with species that face similar information processing constraints. Indeed, there is evidence for shared heuristics and biases between humans and nonhuman primates, such as loss aversion, framing (in which the way choices are posed influences preference), and the endowment effect (in which an item becomes more highly valued once in one's possession) [29], [30], [31]. As yet, however, there is no clear evidence in nonhuman animals for the affect heuristic, in which certain attributes are evaluated while others are neglected, as seen in the less-is-more effect. In one study, macaque monkeys (*Macaca fascicularis*, *mulatta*, *and fuscata*) and a chimpanzee were given a choice between a preferred food versus a combination of the same preferred food and a less preferred one [32]. Rather than finding a less-is-more effect, the investigators found what they called a selective-value effect, in which the subjects were indifferent to the choice alternatives, suggesting that the less-valued item assumed a null value.

In a follow-up study, however, researchers found that chimpanzees are highly sensitive to minute differences in the size of the preferred food, and this sensitivity could explain the chance performance observed in the previous study [33]. When sizes of food items were strictly controlled, the chimpanzees preferred the greater good alone to the combination of the greater and lesser good. This apparent preference, however, was influenced by the intertrial interval (ITI). With a short and variable ITI of simply waiting until the subjects consumed the food, the chimpanzees appeared to maximize their consumption of the preferred food per unit time. To do so, they minimized the time spent consuming the less preferred food. When a constant and relatively long ITI was added (at least 3 minutes), the chimpanzees selected the food combination rather than the preferred food alone [33].

In the end, then, the chimpanzees appeared to respond as humans do when the choice options are compared directly, exhibiting a more ‘rational’ preference for the food combination. However, it is possible that there is an evolutionary progression in the use of the affect heuristic, such that a more distantly-related species may continue to use the heuristic even when making a direct comparison between a greater good and a combination of the greater and a lesser good. Furthermore, because the follow-up study by Beran, Ratliff, and Evans (2009) was conducted only with chimpanzees, the results with macaque monkeys remain inconclusive.

We conducted the current study to determine whether an Old World monkey, the rhesus macaque (*Macaca mulatta*), would show evidence for an affect heuristic by exhibiting a less-is-more effect when evaluating food items. Specifically, we asked whether the monkeys would prefer a highly-valued food item alone over the identical item paired with a food of positive but lower value. We reasoned that if the monkeys preferred the highly-valued food item alone, it would demonstrate the influence of an underlying affect heuristic. First, choice between food items is a quintessential example of an affective evaluation, as the decision is based on the quality and/or quantity of the options [e.g. 13], [14], [15], [16], [19], [21], [34]. Second, if the quantity of the food items were neglected, it would reveal a limitation in the affective evaluation, given that the food combination maximizes reward. Such limitations are diagnostic features of heuristics.

At the same time, it has been argued that any case in which a set or group is represented via an average, prototype, or stereotype may be subsumed under the more general *representative* heuristic, which may be a general information processing strategy for complexity reduction used by the brain to cope with an otherwise intractable world [see 4,9]. Therefore, the demonstration of a less-is-more phenomenon in which a choice option is represented by the quality average rather than sum would also provide some evidence for the shared use of a representative heuristic more generally between humans and a nonhuman animal. The test of the less-is-more effect was conducted twice, under controlled laboratory conditions in Experiment 1 and in the field in Experiment 2.

## Experiment 1

### Methods

#### Ethics statement

Experiment 1 complied with all current laws, regulations, policies, and guidelines of the United States, the United States Department of Agriculture (USDA), the Office of Laboratory Animal Welfare (OLAW), and all procedures were approved by the Institutional Animal Care and Use Committee (IACUC) of Dartmouth College.

#### Subjects

Three male rhesus monkeys were tested: Puck, Hamlet, and Titus, ages 7, 9, and 9 years, respectively. The monkeys were maintained at approximately 95% of their *ad libitum* weights to ensure sufficient motivation and good health, and their diet consisted of primate chow (no. 5038, PMI Feeds Inc., St Louis, Missouri, U.S.A.), supplemented with fresh fruit and vegetables. They were individually housed in a homeroom with automatically regulated lighting (14∶10 hour light∶dark cycle, with lights on at 0600 hours). The facility maintains a full-time animal care and veterinary staff. The monkeys were brought to the testing room in custom-made chairs. The chairs were used to (a) minimize disruption in the test subjects' daily routines, given that they were already acclimated to them from a previous response-time experiment (Knight et al., *under review*); (b) have precise control over the experimental testing conditions, including the timing of the trial sequence; and (c) obtain clear, unbiased choice responses via button presses, with the buttons at fixed positions relative to the monkey on every trial.

#### Materials

In the test room, the monkey and experimenter sat in chairs across the table from each other (distance 76.2 cm/30 inches). The monkey's left arm was loosely restrained, while the right arm was free to make the choice responses. In Condition 1, the monkey made its selection by reaching for the food item presented (see *Procedure* for details). For Conditions 2 and 3, the monkey made its selections by pressing one of two buttons (approximately 16 cm apart measured from the centers) on a panel placed in front of the monkey. We used grapes and vegetables for food items (see below), which were selected (grapes) or cut (half vegetables) to be nearly identical in size and shape to the other same quality items: i.e., all grapes the same, all vegetable pieces the same [see 33].

#### Procedure

To determine appropriate food items for the experiment, we conducted two preliminary conditions before the main test condition. In Condition 1, we identified vegetables that each monkey would eat for 30 consecutive trials: for Hamlet and Titus it was one half of a sugar snap pea; for Puck it was one half of a green bean. We used these vegetables and sizes for the remainder of the experiment. The experimenter sat across the table from the subject and offered it a vegetable for 30 trials, with an ITI of approximately 10 s, which included the time that the monkey took to consume the vegetable.

Condition 2 verified a consistent preference for grapes over vegetables with a two-alternative forced-choice procedure conducted in one 10-trial session. Each trial began when the experimenter placed her hands approximately 10 cm from the back edge of the button panel (from the monkey's point of view) and opened her hands to reveal the alternatives. A grape was in one hand and a vegetable in the other. The position (left or right) of the alternatives was assigned pseudorandomly, with the constraint that the locations did not remain the same for more than three consecutive trials. The experimenter's hands remained in this position until the monkey had looked at the alternatives for approximately 3 s. The experimenter then moved her hands in a straight line toward the monkey, until each food was located directly behind a button. The monkey then made a selection by pressing the button in front of the desired option. The experimenter moved the selected hand toward the monkey while simultaneously closing the other hand, enabling the monkey to retrieve the selected food item. All trials were set up out of view of the monkey, behind an opaque barrier. The ITI was based on consumption time, in that each trial began after the monkey finished consuming the chosen food item. The ITI averages for each monkey were calculated from videotape coding. The average ITI was approximately 22 s for Puck, 10 s for Titus, and 15 s for Hamlet. All three monkeys chose the grape over the vegetable on all ten trials, verifying the strong preference for the grape over the vegetable.

**Figure 1:**
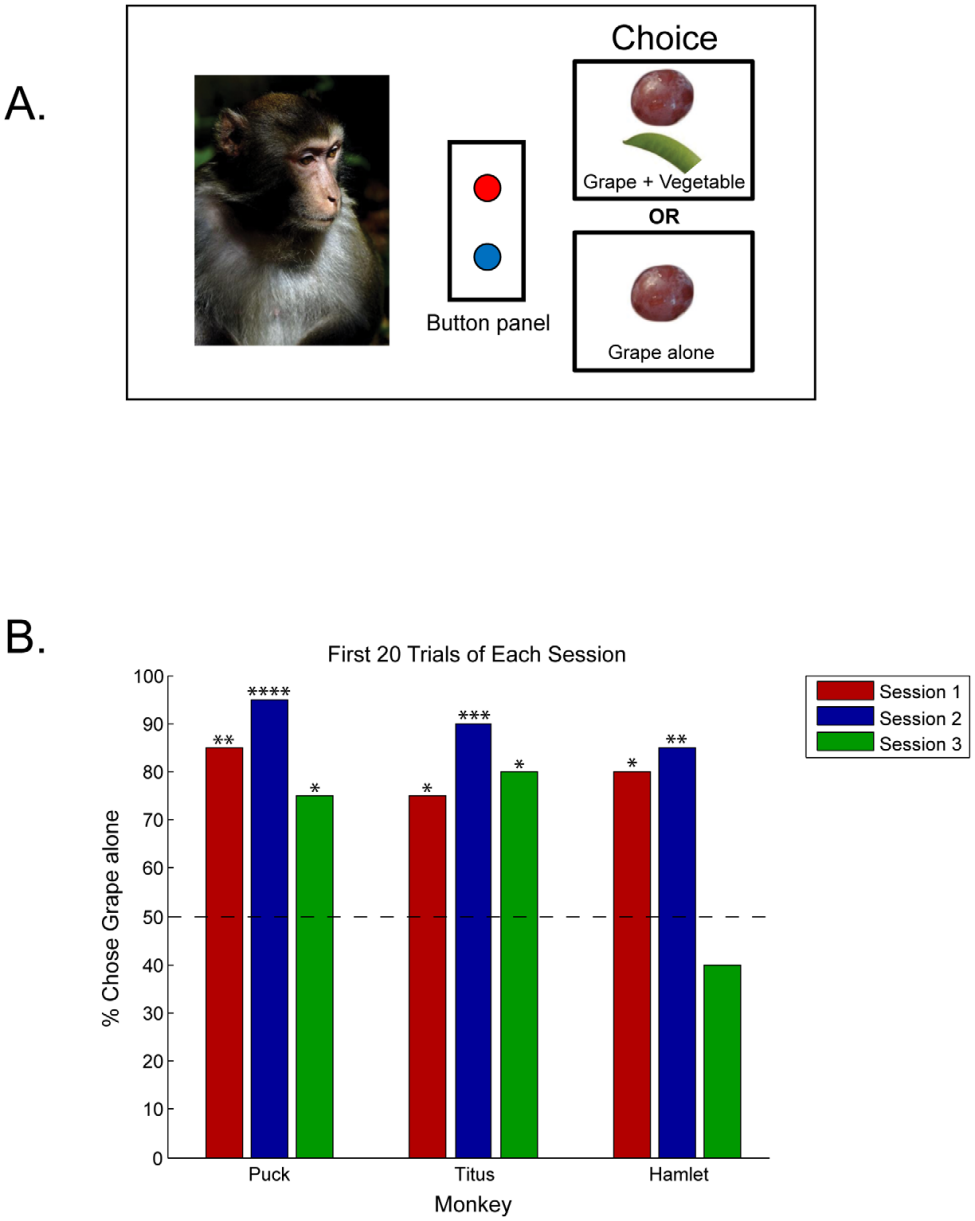
Experiment 1 illustration and results. **A.** Illustration of the main test condition. The monkey pressed the corresponding red or blue button to make a choice. **B.** Results for the first 20 trials of each session for each monkey. Data were analyzed using two-tailed Binomial tests: * P<0.05; ** P<0.01; *** P<0.001; **** P<0.0001.

In the main experimental condition, Condition 3, we tested the monkeys on three 30-trial sessions, conducted on separate days. We used the same procedure as in Condition 2, except that we now placed a grape in one hand and both a grape and the vegetable in the other (Figure 1A). Importantly, we made sure the monkeys obtained both food items when the food combination was selected. That is, if the monkeys appeared to neglect the less-preferred item, the experimenter held the food item in front of the monkey until it was taken. All Condition 3 sessions were videotaped. The ITI was again based on consumption time, with each trial beginning after consumption of the previous trial's chosen food items. After selecting the single grape alone, the average ITI was 21.9 s for Puck, 28.7 s for Titus, and 13.5 s for Hamlet. After selecting grape plus vegetable, the average ITI was 35.4 s for Puck, 49.3 s for Titus, and 21.1 s for Hamlet.

Finally, in Condition 4, to verify that the value of the vegetable by itself remained positive in subjective value, we conducted a final 30-trial test session, in which the experimenter offered the vegetable alone and gave it to the monkey after he touched the corresponding button on the panel. The ITI for this condition was approximately 10 s for all monkeys. Condition 4 was also observed by a second experimenter via a closed-circuit video camera and monitor and videotaped.

To minimize the possible cuing of the subjects by the experimenter, several procedures were implemented, including (a) playing white noise in the test room throughout the session to mask potential auditory cues and distractions; (b) having the experimenter wear a white lab coat, safety glasses, surgical mask and gloves to mask potential visual cues; (c) having the experimenter trained to perform a precise, stereotyped testing procedure; (d) having the experimenter look downward at the center of the testing apparatus and observe the monkey's choices via peripheral vision to avoid providing gaze cues; (e) having a separate individual observe the sessions via closed-circuit camera and monitors; (f) having a separate individual score the results for the main Condition 3 from the video, with perfect correspondence among the experimenter, observers and scorer.

### Results

As stated previously, the fact that all three monkeys accepted and ate the offered vegetable for all 30 trials in Condition 1 shows that the vegetable itself was positive in value. That all of the monkeys selected the grape over the vegetable in all ten trials of Condition 2 verifies that there was a strong preference for the grape. The critical test, then, was Condition 3, in which the monkeys chose between a single grape and a grape plus vegetable option. When averaged over the entire session, the results were mixed. Puck preferred the single grape to the grape plus vegetable in all three test sessions (83%, 93%, 77%; two-tailed Binomial tests: *P* = 0.0003, *P*<0.0001, *P* = 0.0052, respectively); for Titus, although he selected the grape alone more than the grape plus vegetable in every session, there was no significant overall preference in any session (for grape alone: 57%, 67%, 67%); and Hamlet preferred the grape alone in the first two sessions, but not in the third (77%, 80%, 33%; two-tailed Binomial tests: *P* = 0.0052, *P* = 0.0014, *P* = N.S., respectively).

However, in all three sessions, we noted that Titus appeared to satiate on the grape after approximately 20 trials, i.e. at the two-thirds point of the session, and switched his preference from the grape alone to the grape-vegetable combination. Thus, for example, in the first test session, and examining five-trial blocks, Titus' performance was 80% for grape alone on the fourth block and then 0% for grape alone on the fifth block. Across the three sessions, he averaged 82% for the single grape alone in the first 20 trials; and 27% in the final 10 trials. In addition, when he chose the combination, the consumption order generally reversed. In all three sessions combined, the percentage of trials in which the grape was eaten first in the first 20 trials was 100% (11 out of 11), whereas in the last 10 trials it was 36% (8 out of 22). He also took longer to eat the grape in the last 10 trials of every session (average consumption time for the combination in the first 20 and last 10 trials: 34.2 vs. 56.3 s) or discarded it altogether (last three trials of Session 2, last two trials of Session 3).

Therefore, to examine preference before selective satiation, we analyzed the first 20 trials of each session for each monkey. As can be seen in Figure 1B, all of the monkeys preferred the grape alone to the grape plus vegetable in the first 20 trials of sessions one and two (two-tail Binomial tests, Session 1: Puck, 85%, *P* = 0.0026; Titus, 75%, *P* = 0.0414; Hamlet, 80%, *P* = 0.0118; Session 2: Puck, 95%, *P*<0.0001; Titus, 90%, *P* = 0.0004; Hamlet, 85%, *P* = 0.0026). In addition, two of the three monkeys maintained this preference in the first 20 trials of Session 3 (two-tail Binomial tests: Puck, 75%, *P* = 0.0414; Titus, 80%, *P* = 0.0118), while one monkey, Hamlet, exhibited no preference by Session 3.

With respect to actual consumption in Condition 3, the grape was always eaten by all monkeys (on both single grape and combination selections) except for the five aforementioned trials for Titus. When the combination was selected, all monkeys took the vegetable every trial and usually ate it: Puck ate the vegetable 8 out of 14 trials; Titus, 30 out of 33; Hamlet, 29 out of 33. Regarding consumption order, for Puck and Hamlet (Titus described above) the grape was eaten first on all trials, with a single exception during Puck's Session 1.

Finally, in Condition 4, all three of the monkeys accepted the vegetable and ate it on all 30 trials (two-tail Binomial test on each session: 100%, *P*<0.0001).

### Discussion

As seen in Figure 1B, all three monkeys initially preferred the grape alone to the grape-vegetable combination. Thus, at least initially, the overall subjective value for the grape alone was higher than that for the grape and vegetable combination. This effect persisted for two of the three monkeys (Puck and Titus), throughout all three test sessions for Puck, and throughout the first two-thirds of every test session for Titus. The third monkey, Hamlet, initially exhibited a strong preference for the grape alone, but this preference dissipated in the third session. This change in choice behavior is intriguing and suggests that, under certain conditions, rhesus macaques can learn to overcome an initial suboptimal preference.

The preference for the grape alone cannot be explained by a distaste for the vegetable, given that vegetable was selected for the experiment because the monkeys accepted and ate it on all trials in Condition 1; and the vegetable was again accepted and eaten by all monkeys on all Condition 4 trials. Although it is possible that the preference for the grape alone might be explained by a potential taste-taste interaction between the grape and vegetable, such that, in combination, they are distasteful, we find this unlikely. First, we purposely chose fruits and vegetables to minimize a possible taste interaction effect. Second, rhesus monkeys are omnivores and have a wide-ranging palette that includes numerous fruits and vegetables [e.g. 35]. Third, the monkeys in our experiment were food-restricted to maintain a constant level of motivation and good health, and, thus, they should have been motivated to maximize reward. Fourth, when the combination was selected, both were usually eaten. Thus, we think it is unlikely that the reduction in the subjective value of the combination was due to a taste-taste interaction between the food items. Nonetheless, one of the objectives of Experiment 2 was to test the generality of the less-is-more finding using a new pair of food items.

As stated, when the combination was selected, both were typically eaten. In these cases, even though the vegetable reduced the overall combined value, the vegetable itself nonetheless maintained a positive value in the presence of the grape (and *vice versa* for the grape). The results thus provide evidence that the combined effect of the grape and vegetable values resulted in an effective averaging rather than a summation of their individual values. Thus, an averaging heuristic appeared to be applied during valuation in affective decision-making. In contrast, the instances when the monkeys did not eat the vegetable provide evidence for other possible phenomena. For example, the behavior suggests that there are circumstances in which less-preferred food items acquire a null (i.e. selective value effect) [32] or negative value in the presence of the higher-valued item.

The Beran et al. (2009) study suggested that a selective value effect, whereby individuals appear to ignore the less-preferred food item, may have been due to differences in the sizes of the more-preferred food item in the choice options. After using similarly-sized grapes in our study, we also obtained an interaction between the two food items, although rather than the lesser good being ignored, it reduced the overall appeal of the option. In fact, our finding is similar to that obtained with chimpanzees when short and variable ITIs were in place [33]. In this situation, the chimpanzees preferred the single more-preferred piece of banana to a combination of the same banana piece with a less-preferred piece of apple. This preference reversed, however, when a longer ITI was used (at least 3 minutes). When the longer ITI was in place, the chimpanzees chose the food combination over the banana piece alone, thus selecting the ‘optimal’ option with the most food [33]. Thus, it is possible that our results were influenced by the ITI length, and that the same reversal may occur with rhesus macaques with a longer ITI. That all three of the monkeys exhibited a preference for the greater good alone from the onset and that there was no trend over trials toward a greater preference for the single item alone serves as evidence against an ITI effect. Nonetheless, another objective of Experiment 2 was to determine whether the monkeys' preference for the single highly-preferred item was influenced by the repeated trials structure of Experiment 1 that did have an ITI that enabled the monkeys to select and consume the preferred item without any significant pauses.

As has been shown in humans, suboptimal choices often occur with one's immediate, spontaneous reactions and may dissipate as individuals become more familiar with the problem, as we found with Hamlet in Experiment 1. The focus of the current study, however, was not to study the influence of task demands and experience on choice behavior, but rather, to determine if rhesus monkeys spontaneously use an affect heuristic. Thus, in Experiment 2, we sought to test more naïve individuals on only one trial per monkey, in a naturalistic setting, and with a new pair of food items.

## Experiment 2

### Methods

#### Ethics statement

Experiment 2 complied with all current laws and regulations of the United States and Puerto Rico, the United States Department of Agriculture (USDA), and all procedures were approved by the Institutional Animal Care and Use Committee (IACUC) of the Medical Sciences Campus of the University of Puerto Rico.

#### Subjects

Subjects were free-ranging, adult male rhesus monkeys (*Macaca mulatta*) living on Cayo Santiago in Puerto Rico. We tested adult males because (1) it was unclear at the outset how many trials we would obtain, and we attempted to reduce potential variability in the data set due to lack of power; and (2) they were more readily found on the periphery of the social groups, which minimized the potential influence of other monkeys. The colony on this island is owned and maintained by a branch of the Caribbean Primate Research Center of the University of Puerto Rico, and it has approximately 1000 rhesus monkeys living in different social groups. The monkeys live in semi-wild conditions where they are given a daily provision of monkey chow.

#### Food items

We used cucumber (circular cross sections approximately 0.5 cm thick) and 1/16 apple slices, which were cut to be nearly identical in size and shape to the other same quality items: i.e., all cucumber slices the same, all apple slices the same.

#### Testing Procedure

In Condition 1, we conducted a taste test to determine whether the monkeys would eat cucumber slices when they were offered alone, and thus verify that the value of the food item was positive. The experimenter walked around the island searching for individual monkeys to test. When a lone individual was spotted, the experimenter approached to a distance of approximately 1.52–3.05 m (5–10 ft) from the monkey. Next, the experimenter knelt down and placed a white, rectangular Styrofoam tray on the ground. The experimenter then reached into a backpack to obtain a cucumber slice, held it vertically and at chest level for the monkeys to see, then placed the cucumber in the middle of the Styrofoam tray, stood up, and took two to three steps backwards from the tray. This setup procedure took approximately 10 seconds per trial.

In Condition 2, we conducted a preference test with the apples and cucumbers. Two experimenters approached an isolated monkey to a distance of approximately 3.05 m (10 ft). With the monkey as the reference point, the experimenters positioned themselves at an angle of approximately 90°: one 45° to the monkey's left, the other 45° to the monkey's right. This angle allowed the monkey to see both experimenters clearly, while permitting the monkey to approach only one of the two alternatives. Once in position, both experimenters simultaneously knelt on their left knee and placed a white, rectangular Styrofoam tray flat on the ground in front of them. They then reached into their backpacks with their left hands to retrieve the cucumber and apple slices, held up their food option vertically at chest level, waited for the monkey to look at both food options, then placed the food on the tray, stood up, and stepped back from the trays. The entire procedure took approximately 10–15 seconds per trial. Once the experimenters stepped away from the trays, the monkey typically went directly toward one option and was allowed to take the food and eat it.

**Figure 2:**
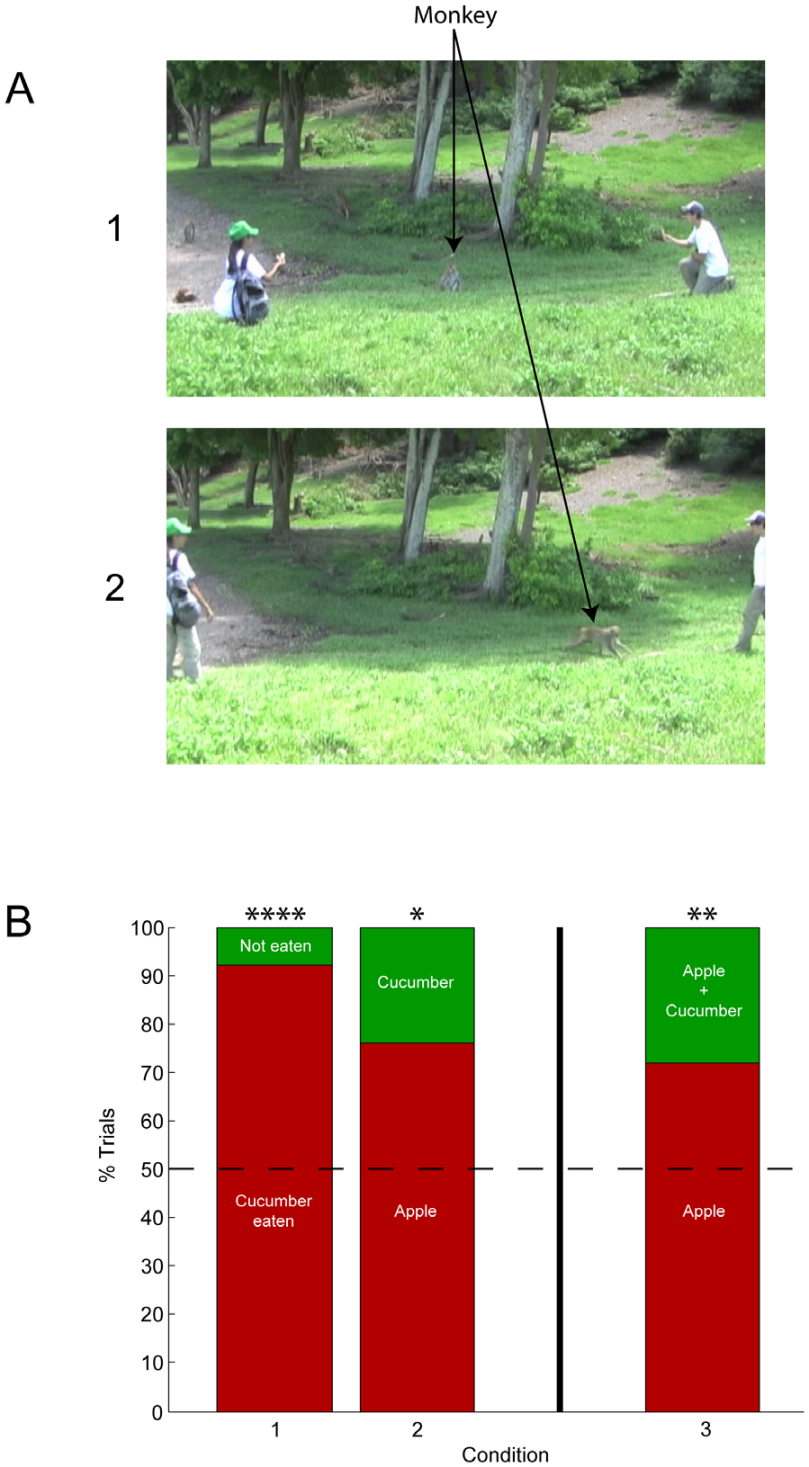
Experiment 2 testing procedure and results. **A**. Depictions of the testing procedure. (1) displaying the choice options to the monkey, with the experimenter on the left (with backpack) presenting the apple and cucumber slices, and the experimenter on the right (backpack not seen in this view), presenting the apple slice alone; (2) the monkey making its choice. **B**. Experimental results for all three test conditions. (1) one cucumber slice alone; (2) a cucumber slice versus an apple slice; (3) an apple and cucumber slice versus an apple slice alone. Data were analyzed using two-tailed Binomial tests: * = P<0.05; ** = P<0.01; **** = P<0.0001.

For Condition 3, the main test condition, we carried out a nearly identical procedure as in the Condition 2 preference test. For this condition, the experimenters offered the monkeys a choice between an apple slice alone or an apple slice plus a cucumber slice (with apple and cucumber slices identical to those in Conditions 1 and 2). When holding up the apple plus cucumber option to display to the monkey, the experimenter held both items simultaneously in the same hand so that the monkey would see both pieces of food clearly. Figure 2A depicts the testing procedure.

Conditions 1–3 were conducted in order, with the first two (taste and preference tests) completed on the same day. The critical test, Condition 3, was conducted on the following day. Conditions 1 and 2 each consisted of 25 trials; Condition 3 consisted of 50 trials. We attempted to minimize possible repeated trials of individual monkeys within conditions, and we utilized the research center's coding system (ear notches and chest tattoos) to identify individuals whenever possible. We also enacted multiple measures to ensure that the experimenters would not affect the outcome, including (a) having two experimenters who were unaware of the specific hypotheses conduct the experiment, (b) with both experimenters wearing white tee-shirts and khaki pants [36], (c) having the experimenters practice the testing routine multiple times to develop a highly consistent, stereotyped and synchronized procedure, (d) making sure the monkeys looked at both choice options and returned to a neutral gaze (i.e. straight ahead) before allowing the monkeys to choose, and (e) not making direct eye contact with the monkeys.

### Results

In Condition 1, in which the experimenter offered a cucumber slice alone, the monkeys approached the tray and consumed the cucumber slice on 92% of the trials (23/25; two-tailed Binomial test, *P*<0.0001; Fig. 2B), including 96% of the identified individuals (22/23; two-tailed Binomial test, *P*<0.0001). This result shows that the cucumber slice generally had a positive value when offered alone.

In Condition 2, in which two experimenters offered the monkeys a choice between one apple slice and one cucumber slice, the monkeys chose the apple slice in 76% of the trials (19/25; two-tailed Binomial test, *P* = 0.0146; Fig. 2B), including 77% of the identified individuals (17/22; two-tailed Binomial test, *P* = 0.0169), demonstrating that, on average, the monkeys preferred the apple slice to the cucumber.

In the main test, Condition 3, the monkeys were offered either one apple slice or an identical apple slice with an additional cucumber slice (Figure 2A). The monkeys chose the apple slice alone in 72% of the trials (36/50; two-tailed Binomial test, *P* = 0.0026; Figure 2B), including 74% of the identified individuals (31/42; two-tailed Binomial test, *P* = 0.0029).

### Discussion

Compared to Experiment 1, this experiment was conducted in a more naturalistic setting, with many more individuals, with two different food items, and with a single trial design that precluded any possible repeated-trials influence on choice behavior in Condition 3. Despite these differences, the experiments yielded the same general result. Again, the monkeys preferred the greater good alone (apple slice) to the greater and lesser good combination (apple and cucumber slice).

It is possible that the monkeys viewed the choice options as risky, and perhaps assumed they would likely obtain only one of the items, and thus approached the lone apple slice. However, we do not think this is likely. First, following the same logic, it appears to us that the two-item option would provide a better opportunity to obtain at least one of them. Second, other studies using the same general testing paradigm showed that when choosing between different quantities of the same food item (e.g. apple slices), the monkeys chose the larger quantity (after factoring out discounting due to distance from the monkey) [37]. Nonetheless, we do think that how the problem is posed could influence how the food items are evaluated. For example, when choosing in isolation, i.e. without the experimenters present, it is possible that monkeys could show more of an indifference to the two options, i.e. a selective-value effect [32], given that the lower-valued option could simply be neglected without any cost to the individual. To be sure, the specific conditions under which different effects may occur must be further investigated.

Finally, although it is possible that the monkeys preferred the apple slice alone due to a distaste for the apple-cucumber combination, we again believe this is unlikely for reasons enumerated in the Discussion section of Experiment 1, including the wide-ranging diet of rhesus macaques, which includes fruits and vegetables [35]. Moreover, because we obtained the same findings in Experiment 1Experiments 1 and 2 with different food items, it is less likely to be due to the specific food combinations.

## General Discussion

When given a choice between a greater good alone versus the greater good together with a lesser one, the monkeys in Experiment 2 preferred the greater good alone, as did all three monkeys at the onset of Experiment 1, a bias that persisted for two of the three Experiment 1 monkeys. The preference occurred even though the higher-quality food was included, in its entirety, in the greater offering. To maximize utility, the monkeys should have chosen the combination. Because (a) the monkeys in Experiment 1 exhibited a preference for the greater good alone from the onset, (b) there was no trend over trials in Experiment 1 toward a greater preference for the single item alone; and (c) the single-trial design of Experiment 2 precluded a potential repeated-trials effect on preference in Condition 3, our results provide experimental evidence that rhesus macaques sometimes prefer less food to more food.

This finding contrasts with work showing that animals, including rhesus macaques, have a strong prepotent bias against selecting the smaller of two quantities of food [38], [39], [40], [41], [42], [43], [44], [45], [46], [47]. Although contrast effects have been studied extensively in nonhuman animals [see 32,48,49,50,51], we believe this is the first report of a nonhuman animal spontaneously choosing the lesser of two food amounts simply due to the evaluation of the food items, with all items being positively valued. In other cases that have reported a preference for less food, social factors appear to override choice preferences. Some nonhuman primates have turned down offers when another individual receives a better one [52], [53] or when offered something better than a social partner [53].

In this study, the results appear to reflect an evolutionarily-conserved process that reflects how choice options are sometimes evaluated when multiple dimensions (e.g. quantity and quality) vary. Rather than summing the individual and independent subjective values of the items in each set, the monkeys appeared to neglect quantity and assessed the choices based on quality, with the lower-valued food item reducing the attractiveness of the aggregate option. Because the monkeys in our study made choices based on a small number of food items in view, and because their choices reflected a quality averaging rather than summation leading to a suboptimal decision, our results suggest that under at least some conditions rhesus macaque choice behavior reflects the use of an underlying affective evaluation heuristic: i.e. an affect heuristic. Further work will need to determine the extent to which this heuristic is applied, especially since it has been argued that affect and emotion likely play a primary and ubiquitous role in information processing, helping to reduce the processing load in complex environments [4], [5], [11], [12], [13], [14], [15], [16], [17], [18], [19], [20], [21], [22], [23].

At the same time, affective evaluation processes utilize perceptual and cognitive mechanisms, the relative contributions of which have yet to be determined in the context of the less-is-more effect (Hsee, et al., 1999; Hsee & Zhang, 2004; Hsee & Zhang, 2010; Kahneman, 2003, 2011; Slovic, et al., 2002). In fact, our finding could reflect an information processing strategy for complexity reduction that goes beyond affect. For example, it has been argued that the more general *representative* heuristic may subsume all cases in which members of a set, category or group (e.g. objects, people, events) are represented by an average, prototype, stereotype, or schema (Kahneman, 2003, 2011; Slovic, et al., 2002; Kahneman, Slovic, & Tversky, 1982). In that regard, our finding that an average replaced a summation of individual values provides evidence for the conservation of the representative heuristic more generally. In any event, further work is required to determine the pervasiveness of the heuristic, and whether it stems from one or multiple underlying sources. Certainly, other related phenomena are likely to be involved, including *evaluability*, in that the effect is likely influenced by the ease of evaluation of the relevant dimensions [4], [9], [26], [27], [28], [54]. Thus, as discussed in the introduction, it has been argued that dimensions such as quantity are more difficult to evaluate (e.g. how good is two of something) than others such as quality (e.g. how good would a food item taste). Our results support this claim, given that the monkeys' choices reflected a dominance of quality over quantity in their evaluations.

Although the affect heuristic in humans could result from the need to cope with the complexities of human societies, such as the rapidly changing aggregations of goods and costs in our complex trading culture, this study's findings suggest that it may instead be an evolved adaptation shared by the two species. Thus, the heuristic may have evolved in or before the last common ancestor of catarrhine primates, a group that includes all Old World monkeys, apes, and humans. Alternatively, this behavior may have evolved independently due to convergent selection pressures in the rhesus macaque and human lineages.

Despite the similarity in these biases in monkeys and humans, there are important differences [55]. Although people clearly exhibit ‘less-is-more’ irrationality, it thus far has been shown in four main contexts. First, it occurs when direct contrasts are precluded and the ‘less’ and ‘more’ alternatives are evaluated in isolation from each other [1], [24], [26], [27], [28]. Second, it occurs when value is assessed over time, such as when items are received sequentially [56], [57], [58], [59], [60], [61], [62]. In these cases, duration is neglected and people exhibit a preference for alternatives that end well (i.e. a peak-end effect) over ones that simply have the highest overall aggregate value over time. In the extreme, people actually prefer a longer duration of pain if it subsides over time (rather than a shorter duration that remains constant) [56], [57], [58], [59]. Third, it occurs when ‘less’ and ‘more’ alternatives have different degrees of predictability, with positive outcomes that were unexpected or uncertain being more pleasurable than expected ones even of objectively higher value [e.g. 9], [63]. Finally, the fourth context regards the more general representative heuristic and occurs when an individual believes that the *less* likely of two hypothetical options is actually *more* likely. For example, Kahneman and Tversky have famously shown that when people are told that a woman was a liberal activist in her past, most think it is more likely that the woman is both a bank teller *and* an active feminist than a bank teller, even though the latter includes the former and therefore must be more probable (i.e. the “Linda” problem) [4], [9].

In fact, like humans, chimpanzees also appear to make the optimal choice (the higher aggregate value) when the options are presented simultaneously, enabling direct contrasts [33]. In our study, the monkeys made suboptimal choices with the options presented simultaneously. Thus, although rhesus macaques and humans share an affect heuristic, higher-level processes that can override the heuristic (e.g. executive processes of the prefrontal cortex) may be superior in humans and hominids more generally (i.e. great apes and humans) [4], [10], [27], [64], [65], [66], [67], [68], [69], [70]. It will be important to continue to delineate the similarities, differences, and evolutionary trajectory of the underlying processes used by different species. For example, other species may share the capacity, but require additional experience to reach the same levels of performance. Indeed, the less-is-more effect dissipated for Hamlet in Experiment 1. Thus, rhesus macaques have the capacity to override the heuristic, and with experience, they may learn to make ‘rational’ choices, in at least some situations. At the same time, the direct comparison of the choice options in our experiments might represent an extreme test of the phenomenon and one that might be easier to overcome.

In summary, we have found that rhesus macaques, like people, decide between choice items using an affect heuristic, which sometimes results in suboptimal choices. This heuristic may be advantageous in complex, uncertain, competitive, and otherwise time-sensitive environments. If time is of the essence, fast approximate evaluation processes will outcompete slower, more deliberative ones. Furthermore, under many foraging conditions, the heuristic may lead to near optimal decisions. First, when food is plentiful, using average quality would maximize outcome. Second, when food is less plentiful, food type — that is, quality — at a given site (such as a particular fig tree) might be a more reliable attribute to track than others, such as quantity or duration. Thus, choices among food patches that contain different types of resources (e.g. different fruits, vegetables, or nuts) may be strongly influenced by average quality comparisons. These possibilities are supported by findings in which foraging primates bypass relatively abundant lower-quality food items for higher-quality ones that are farther away or scarcer [e.g. 71], [72], [73]. Finally, even for a given food type, the highest-quality items might be especially preferred. This is supported by findings that have shown, for example, that relatively prosocial capuchin monkeys foraging in a given tree are willing to fight for the outside positions that contain the highest-quality fruits more exposed to sunlight rather than settle for the larger number of lower-quality fruits toward the tree crown center [74], [75].

Thus, an affect heuristic may provide important selective advantages. The downside of the adaptation, however, is irrational decision-making with respect to potentially important attributes that are neglected, such as quantity. This limitation might reflect selection pressures that led to information processing systems that are good enough rather than optimal. At the same time, the affect heuristic — like other heuristics and biases — is likely the means by which natural cognitive systems solve challenging problems in complex, uncertain, and time-sensitive environments [4], [5], [6], [7], [9], [11]. Such suboptimal behavior indicates that, for the brain at least, less can be more.
